# Base editing with high efficiency in allotetraploid oilseed rape by A3A‐PBE system

**DOI:** 10.1111/pbi.13444

**Published:** 2020-08-04

**Authors:** Hongtao Cheng, Mengyu Hao, Bingli Ding, Desheng Mei, Wenxiang Wang, Hui Wang, Rijin Zhou, Jia Liu, Chao Li, Qiong Hu

**Affiliations:** ^1^ Oil Crops Research Institute of Chinese Academy of Agricultural Sciences Key Laboratory for Biological Sciences and Genetic Improvement of Oil Crops Ministry of Agriculture and Rural Affairs Wuhan China

**Keywords:** *Brasscia napus*, base editing, cytidine deaminase, A3A‐PBE, off‐target, herbicide resistance, semi‐dwarf

## Abstract

CRISPR/Cas‐base editing is an emerging technology that could convert a nucleotide to another type at the target site. In this study, A3A‐PBE system consisting of human A3A cytidine deaminase fused with a Cas9 nickase and uracil glycosylase inhibitor was established and developed in allotetraploid *Brassica napus*. We designed three sgRNAs to target *ALS*, *RGA* and *IAA7* genes, respectively. Base‐editing efficiency was demonstrated to be more than 20% for all the three target genes. Target sequencing results revealed that the editing window ranged from C1 to C10 of the PAM sequence. Base‐edited plants of *ALS* conferred high herbicide resistance, while base‐edited plants of *RGA* or *IAA7* exhibited decreased plant height. All the base editing could be genetically inherited from T_0_ to T_1_ generation. Several Indel mutations were confirmed at the target sites for all the three sgRNAs. Furthermore, though no C to T substitution was detected at the most potential off‐target sites, large‐scale SNP variations were determined through whole‐genome sequencing between some base‐edited and wild‐type plants. These results revealed that A3A‐PBE base‐editing system could effectively convert C to T substitution with high‐editing efficiency and broadened editing window in oilseed rape. Mutants for *ALS*, *IAA7* and *RGA* genes could be potentially applied to confer herbicide resistance for weed control or with better plant architecture suitable for mechanic harvesting.

## Introduction

Single nucleotide change is usually associated with agronomic traits and was applied as the important target for genetic improvement of crops (Henikoff and Comai, 2003; Zhang *et al*., 2018). Amino acid substitution or stop codon caused by single nucleotide mutation may lead to losing or changing of protein function. Plenty of mutants created by traditional mutagenesis methods, such EMS and physical radiation, have been implemented in crop genetic improvement (Voytas and Gao, 2014). However, these methods generated lots of random mutations and required intensive labour force to identify the exact mutation type. During the past decade, genome editing technology has been widely applied in modification of desired agronomic traits. Class type II CRISPR‐Cas9 system was most frequently utilized for genome editing in different species (Chen *et al*., 2019; Komor *et al*., 2017; Li *et al*., 2018a; Mao *et al*., 2017). In the CRISPR‐Cas system, endonuclease Cas9 (or Cas12a or Cas12b) can target to specific DNA sequence under the guidance of sgRNA (or crRNA). Then, DNA double‐stranded break is generated (Teng *et al*., 2018; Zetsche *et al*., 2015; Zhang *et al*., 2020). DNA repairing process is mainly occurred through the non‐homologous end joining pathway. Thus, mutations including random insertion or deletion at the cleavage site could be introduced (Carroll, 2014; Zhang *et al*., 2018). Therefore, this system always leads to loss of function of target genes. Base editors are recently developed technology, based on CRISPR‐Cas9 system and cytidine deaminase or adenine deaminase, which do not require double‐DNA‐stranded breaks and can enable specific base conversion at the target site (Gaudelli *et al*., 2017; Komor *et al*., 2016). Base‐editing method has become a promising tool for precise genetic modification of important agronomic traits (Chen *et al*., 2019; Li *et al*., 2020; Manghwar *et al*., 2019).

Two base editors, cytosine base editor (CBE) and adenine base editor (ABE), have been exploited in plants to introduce targeted C to T and A to G mutations at specific genome site, respectively (Chen *et al*., 2019). Base editor system CBE consists of cytidine deaminase APOBEC1, Cas9 nickase (nCas9) and uracil glycosylase inhibitor (UGI). CBE system was the first developed base‐editing toolbox that can converts C to T at specific target site with sgRNA and has been applied in several species (Li *et al*., 2017; Lu and Zhu, 2017; Qin *et al*., 2019a; Shimatani *et al*., 2017; Zong *et al*., 2017). However, the base‐editing scope of CBE system was restricted in narrow target range and the editing efficiency was still considerably low (Hua *et al*., 2019). Recently, A3A‐PBE consisting of human APOBEC3A cytidine deaminase and Cas9 nickase was demonstrated to be more efficient in converting C to T mutations with larger base‐editing window (Zong *et al*., 2018). The other base‐editing system ABEs, consisting of the adenine deaminase TadA from *E*. *coli* and Cas9 nickase or nuclease dead Cas9, were developed to convert targeted A to G in a programmable manner (Gaudelli *et al*., 2017). The ABEs containing ecTadA‐ecTadA*7.10‐nSpCas9 (D10A) fusion have been adopted to perform adenine base editing in plant though the editing efficiency was relatively low especially in specific crops (Hua *et al*., 2018; Kang *et al*., 2018; Li *et al*., 2018b; Suhas *et al*., 2020; Wang *et al*., 2019; Yan *et al*., 2018).

*Brassica napus* (AACC) is an allotetraploid derived from hybridization of *Brassica rapa* (AA) and *Brassica oleracea* (CC) (Chalhoub et al., 2014). Most genes in *B*.*napus* (AACC) possess multiple copies with high sequence similarity which hinders the characterization of gene function. The development of genome sequencing promotes functional genomics study in *B*.* napus* (Sun *et al*., 2017). CRISPR/Cas9 genome editing system has been applied in oilseed rape. Genes controlling important agronomic traits, such as pod shattering, plant height, seed coat colour and silique locular, have been modified using CRISPR/Cas9 method (Li *et al*., 2018c; Yang *et al*., 2017; Yang *et al*., 2018; Zaman *et al*., 2019; Zhai *et al*., 2019 and 2020; Zheng *et al*., 2020 ). One base‐editing system, which is consisted of nCas9, rat cytidine deaminase and a uracil glycosylase inhibitor, has been established in Brassicaceae (Wu *et al*., 2020). Four plants of *BnaC01*.*ALS* were detected to contain effective base variation at the target site, and the editing efficiency was approximately 1.8% (Wu *et al*., 2020).

In this study, we established a newly developed A3A‐PBE system consisting of the human A3A cytidine deaminase fused with a Cas9 nickase and uracil glycosylase inhibitor, and demonstrated that this system was effective in generating C to T mutation at the target site in oilseed rape. Herbicide resistance and dwarf architecture are two important agronomic traits for mechanic production in oilseed rape. Specific point mutations of *ALS* genes confer sufficient tolerance to several kinds of herbicides. Both mutations of the conserved motif (GWPPV) of *IAA7* genes and the conserved motif (VHYNP) of *RGA* genes lead to dwarf phenotype in oilseed rape (Cheng *et al*., 2019; Li *et al*., 2019; Zhao *et al*., 2017; Zhao *et al*., 2019; Zheng *et al*., 2019). Thus, these genes were selected and corresponding sgRNAs were designed for base editing to create genetic materials for the genetic improvement of *B*.* napus*. We achieved an efficiency of over 20% for the three target genes, respectively, suggesting that A3A‐PBE system is effective in C to T conversion at the targeted sites. Our study has established an efficient and precise base editor system A3A‐PBE to facilitate the creation of mutants with herbicide‐resistant and semi‐dwarf architecture, both being important traits for the mechanization of rapeseed production.

## Results and discussions

### **Different sgRNAs were designed for base editing in *B***.***napus***

**Figure 1:**
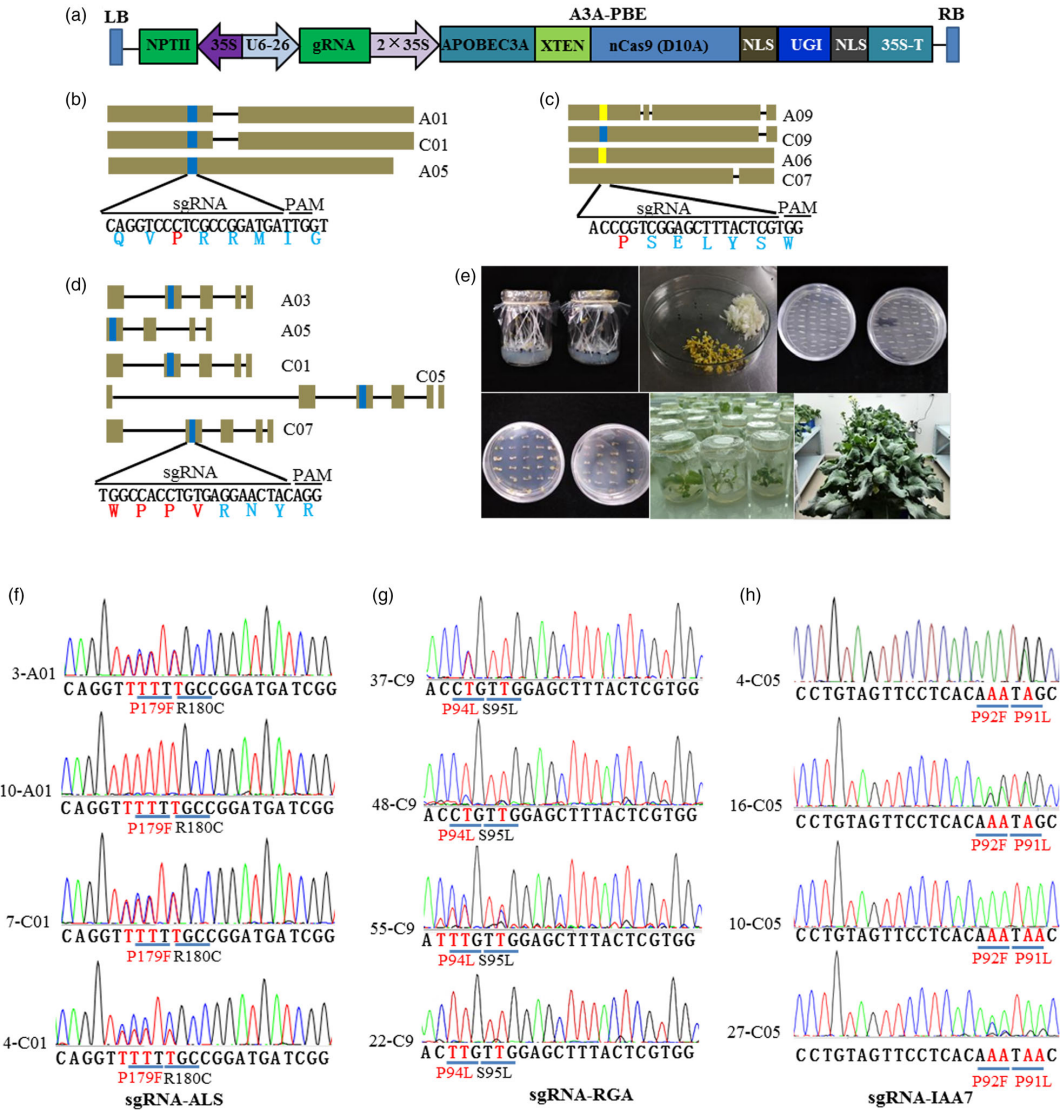
Vector construction and target site mutation analysis of A3A‐PBE system in oilseed rape. (a) Schematic diagram of the vector element of A3A‐PBE. (b) to (d), Schematic view of sgRNA1 target sites for the homoeologs of *ALS*, *RGA* and *IAA7* genes, respectively. The target sequences are highlighted in blue. The orange represented sgRNA with one or two nucleotide acid mismatch. (e) Agrobacterium‐mediated genetic transformation processes in oilseed rape, including hypocotyls elongation, callus induction, plant regeneration, rooting and transfer of transgenic plants to greenhouse. f‐h, Chromatograms of Sanger sequencing results for the sgRNA sites of *ALS*, *RGA* and *IAA7* genes. The base substitution sites are highlighted by red colours. Amino acid conversion was indicated under the target sequence.

We previously demonstrated that AtU6‐26 promoter driving the sgRNA transcription system could achieve high genome editing efficiency in *B*.* napus* (Li *et al*., 2018c). To establish high base‐editing efficiency system in *B*.* napus*, A3A‐PBE elements and sgRNA were driven by 2 × 35S and AtU6‐26 promoters, respectively (Figure 1a). Different sgRNAs were devised to target *ALS*, *RGA* and *IAA7* genes in oilseed rape, respectively. Five homoeologs of *ALS* were identified in *B*.*napus* genome, and three of them have been verified to be functionally expressed genes, including *BnaA01*.*ALS* (*BnaA01g20380D*), *BnaC01*.*ALS* (*BnaC01g25380D*) and *BnaA05*.*ALS* (*BnaA05g03070D*) (Ogulle et al., 1992). We then designed one sgRNA‐matched well with all the three functional homoeologs of *BnaALS* (Figure 1b). After sequence alignment, no conserved sgRNA could be designed to match well with all four homoeologs of *BnaRGA* gene. We then selected one sgRNA which could well target *BnRGA*.*C09* (*BnaC09g52270D*) but with one to three bases mismatched with the other *RGA* homoeologs (Figure 1c). For *IAA7* genes, five copies were recognized after checking genome sequence of *B*.* napus*. One conserved sgRNA was designed to target all the five copies after sequence alignment (Figure 1d). Three constructs were independently transformed into ZS6 via Agrobacterium‐mediated transformation (Figure 1e). Several independent T_0_ transgenic plants from different constructs were generated. After PCR detection by *nCas9* and *NPTII* gene‐specific primers, we obtained 38, 63 and 32 positive transformants from sgRNA‐*ALS1*, sgRNA‐*RGA* and sgRNA‐*IAA7*, respectively (Table 1). All these positive transgenic plants were then analysed for target base editing.

**Table 1:** Base‐editing efficiency of *BnaALS*, *BnaRGA1* and *BnaIAA7* genes

Target gene	Location	sgRNA	Number of transgenic plants	Number of plants with base editing	Base‐editing efficiency (%)
*BnaALS*	*BnaA01g20380D*	sgRNA1	38	9	23.68
	*BnaC01g25380D*			8	21.05
*BnaRGA*	*BnaC09g52270D*	sgRNA2	63	16	25.39
	*BnaA09g18700D*			2	3.17
*BnaIAA7*	*BnaA03g36950D*	sgRNA3	32	10	31.25
	*BnaC05g29300D*			7	21.87
	*BnaC01g43640D*			10	31.25
	*BnaA05g16680D*			10	31.25

### Target mutation analysis of transgenic plants for *ALS* gene

To explore the viability and efficiency of A3A‐PBE system in oilseed rape, positive transgenic plants of three constructs were examined by Sanger sequencing. It was shown that 11 out of 38 positive plants for *ALS* contained C to T conversion in the editing window of sgRNA target region. Nine and eight plants were identified to have C to T conversion at *BnaA01*.*ALS* and *BnaC01*.*ALS* target site, respectively (Table 1 and Table S2). Six transgenic plants were identified to harbour C to T conversion in both *BnaA01*.*ALS* and *BnaALS*.*C01* target sites. Of the 11 mutant plants, 8 plants harboured heterozygous mutation in *BnaALS*.*C01* target site in T_0_ generation (Table S2). Three plants were identified to be homozygous mutants, while other 7 plants were heterozygous mutants in *BnaA01*.*ALS* target site in T_0_ generation (Table S2). Among these plants with base editing, five mutation types were identified in the editing window (Figure 1f, Table S2). Base substitution (C to T) at C7 and C8 can lead to Pro to Phe amino acid change and will confer herbicide resistance. The plants harboured substitutions at C1, C6, C7, C8 and C10 sites, indicated that the deamination editing window could range from 1 to 10 base sites. As BE3 system was demonstrated to efficiently edit single base within approximately a five‐nucleotide window of −16 to −12 bp from the PAM sequence (with deamination editing window from C4 to C8) (Wu *et al*., 2020), our results showed A3A‐PBE system is more efficient to conduct base editing in allotetraploid *B*.* napus*. Among all the 11 edited plants of *BnaALS*, each one harboured at least three site base substitutions simultaneously. However, it should be noted that wide editing window may be also a limitation of DNA base editors, which causes base variation beyond target sites. High‐precision base editors with narrowed editing window are needed to overcome this limitation (Suhas *et al*., 2020).

Though we have detected C to T conversion in both A01 and C01 chromosome, no base editing was observed in A05 chromosome. Previous results showed that the expression of *BnaA05*.*ALS* was only enriched in the reproduction organs (Ouellet *et al*., 1992). There have no *BnaA05*.*ALS* mutant plants been reported until now, neither by EMS or other technologies. We speculated that base editing of conserved region of *BnaA05*.*ALS* may lead to plant lethality. Besides the expected base‐editing mutations in the editing window, we simultaneously discerned two edited plants with base conversion at target site also harboured allelic Indel mutation or G to T conversion at the target site (Table S2).

### Mutation of *ALS* gene conferred herbicide resistance

**Figure 2:**
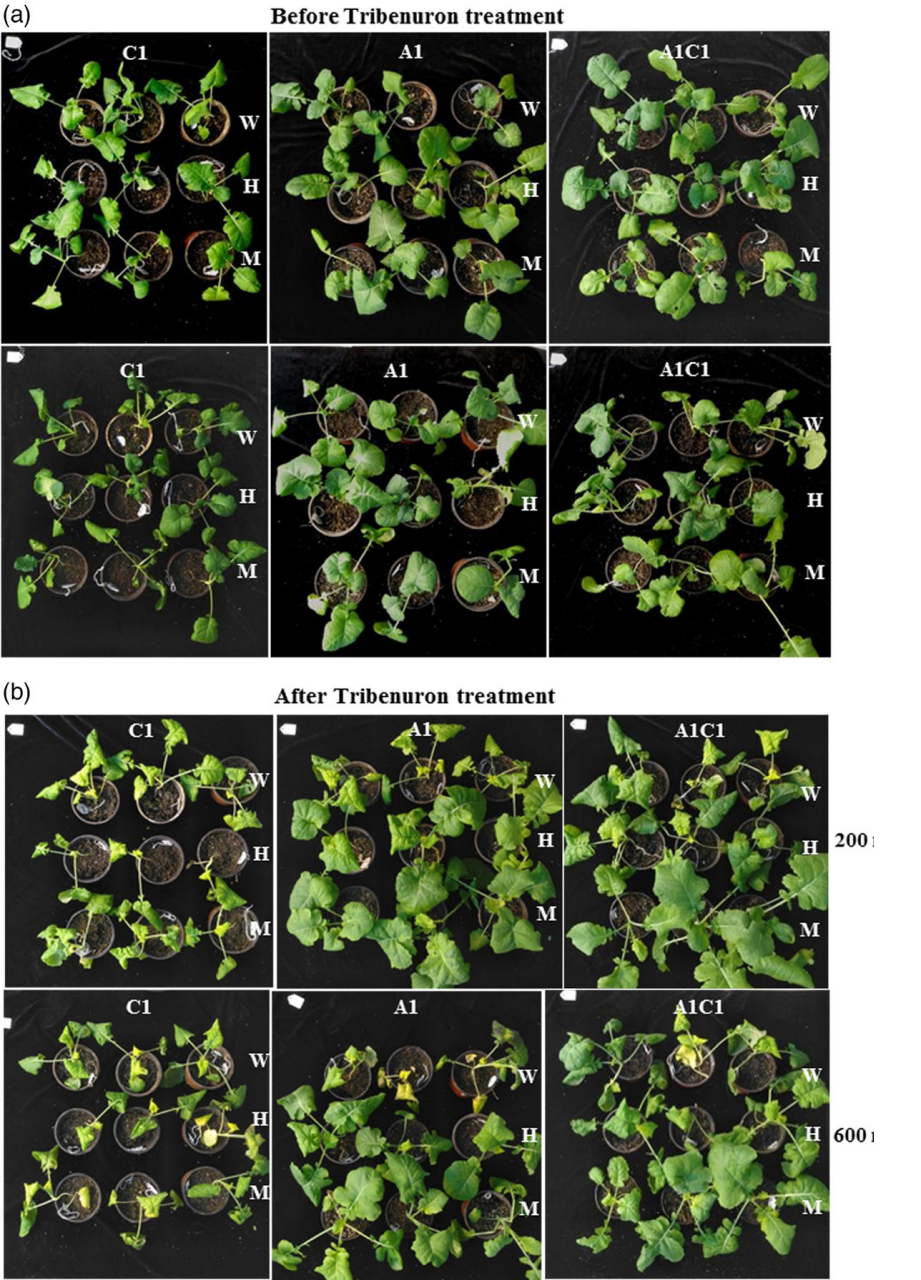
Herbicide resistance detection of the *ALS* base‐edited plants. (a) and (b), Phenotypes of base‐edited plants with different homoeologs mutation and wild type before and after being exposed to sulphonylurea herbicide tribenuron at 200 mg/L and 600 mg/L concentration. C1 and A1 represent plants with base editing of *ALS* in C01 or A01 chromosome derived from *ALS4* and *ALS25* T_0_ plants, respectively. A1C1 represented plants with base editing in both C01 and A01 chromosomes derived from *ALS18* T_0_ plant. W, wild type; H, heterozygous mutants; M, homozygous mutants.

Acetolactate synthase (*ALS*) is the first enzyme for the biosynthesis of branched‐chain amino acids including leucine, valine and isoleucine (Powles and Yu, 2010; Vila‐Aiub *et al*., 2009). Point mutations of *ALS* genes could confer sufficient tolerance to several kinds of herbicides with little damage to plant growth. Amino acid substitution P197F (numbered according to ALS sequence in Arabidopsis) enables various plants to confer herbicide resistance (Chen *et al*., 2017; Tian *et al*., 2018; Zhang *et al*., 2019). However, improper mutation of *ALS* may also disturb gene function and stop the synthesis of branched‐chain amino acid and thus leads to plant death. To detect whether point mutation of *ALS* gene in *B*.*napus* could endow herbicide tolerance sufficiently, T_1_ families from T_0_ plants (*ALS4*, *ALS18* and *ALS25*) was isolated with base conversion of different homoeolog combination and sprayed with sulphonylureas herbicide at two concentrations. After exposure to tribenuron with 200 mg/L (with 10% effective concentration) for 14 days, wild‐type and negative plants without base mutation were growth injured with yellow leaves (Figure 2a and b). The damage to plant growth was even more serious at the 600 mg/L tribenuron treatment (Figure 2b). However, plants with base conversion either in the A1 or C1 chromosome exhibited better growth status at the 200 mg/L concentration (Figure 2b). Both heterozygous and homozygous mutants of *BnaA01*.*ALS* conferred better resistance to 200 or 600 mg/L herbicide treatment compared with wild type. However, plants with base mutation in only *BnaC01*.*ALS* also displayed serious damage after 600 mg/L herbicide treatment (Figure 2b). Therefore, base conversion in the target site of *BnaALS* homoeologs may confer herbicide resistance with different effects. T_1_ families with base conversion in both homoeologs of *BnaALS* gene were then isolated to further investigate herbicide resistance. When exposed to high concentration tribenuron (with 600 mg/L), plants with two alleles simultaneously mutated grew well whereas plants with only one allele mutated alone (*BnaA01*.*ALS* or *BnaC01*.*ALS*) exhibited partially growth stunted or leaf injury (Figure 2b). Our results suggested that mutants pyramiding more resistance alleles could address better sulphonylurea herbicide resistance for weed control in *B*.* napus*. The herbicide resistance conferred by mutation of *ALS* was in a manner of dose effect.

### Target mutation analysis of transgenic plants for *RGA* and *IAA7* genes

Diversiform dwarf mutant is crucial germplasm for lodging and fertilizer resistance, thus is effective for mechanic harvesting of crops. Amino acid variation in the conserved motif (GWPPV) of *IAA7* or conserved motif (VHYNP) of *RGA* genes resulted in significantly decreased plant height. For *RGA* gene in oilseed rape, sgRNA was only fully matched with one homoeolog (*BnaC09g52270D*). It was shown that 16 plants from 63 positive transgenic plants contained base conversion with C to T at the target site, with editing window at C2, C3, C4 and C7 (Figure 1g, Table 1 and Table S3). Eight plants were identified to be homozygous mutants for the target gene. Five plants were also found to harbour base deletion at the target site (Table S3). As the sgRNA mismatched *BnaA09g18700D* with one base, we then checked the base conversion at this target site. Only 2 plants were found to harbour base conversion with C to T, but also with G to T and T to C base conversion (Table 1 and Table S3). From 32 positive plants of *BnaIAA7* gene, 10 plants harboured mutation at target site from different *BnaIAA7* homoeologs (Figure 1h, Table 1). Seven plants contained base mutation in all five *BnaIAA7* homoeologs, while other three plants harboured mutation in three homoeologs. Two plants *IAA10* and *IAA23* contained homozygous mutations in four homoeologs at the target site. Four plants harboured homozygous mutations in at least two homoeologs. Among these plants, four mutation types were identified with the editing window at C3, C4, C6 and C7 site (Table S4). Finally, the base‐editing efficiency was more than 20% for all of three targeted genes (Table 1). Although this editing efficiency is quite high in allotetraploid oilseed rape, it is still relatively low compared with those in Arabidopsis and rice. Promoters driving A3A‐PBE and sgRNA need to be modified to further increase editing efficiency, such as using native U6 promotor from oilseed rape instead of AtU6‐26.

### Specific mutation in *IAA7* or *RGA* conserved domain leads to dwarf phenotype

**Figure 3:**
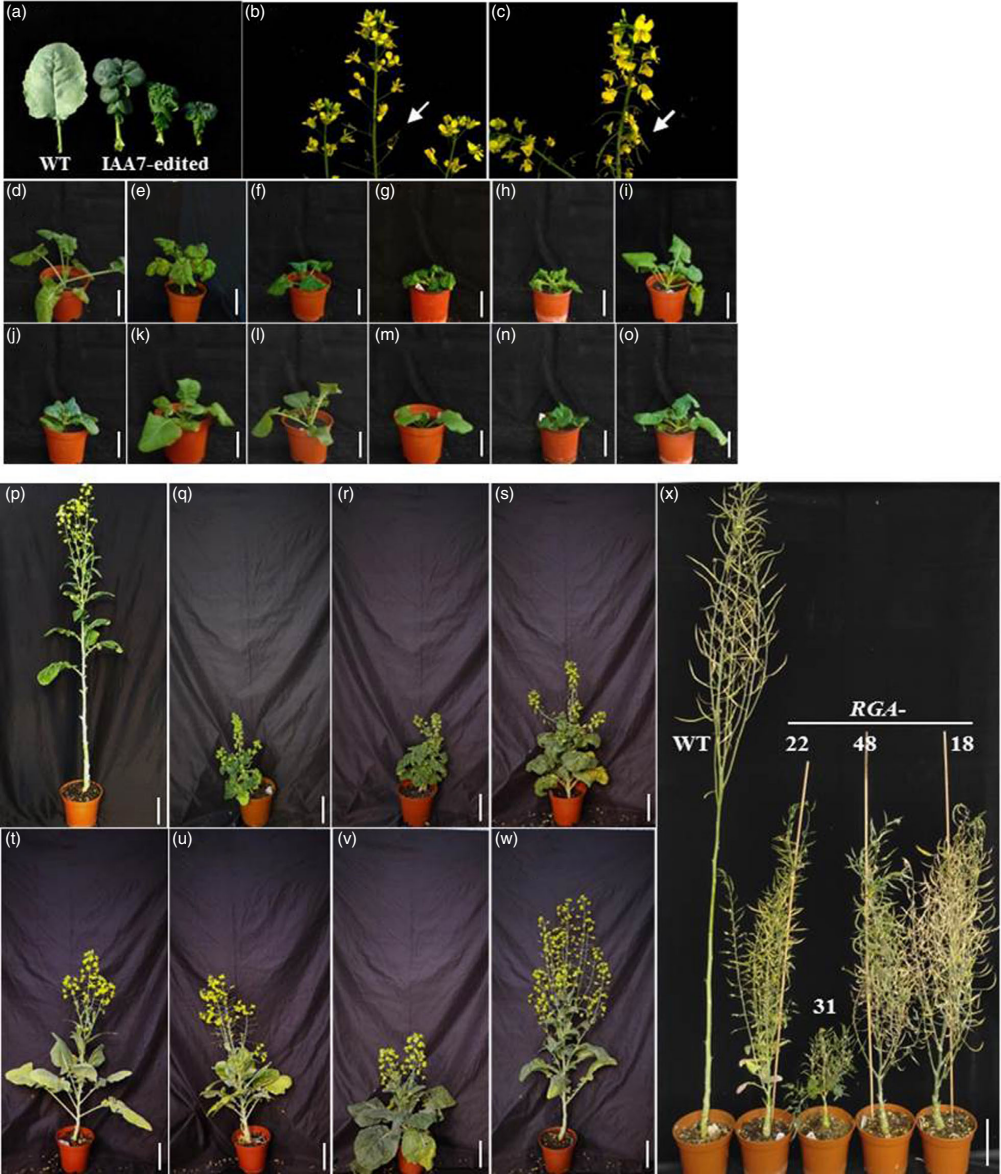
Phenotypes of *RGA‐* and *IAA7‐*base‐edited plants at seedling and maturation stage. (a) Leaves observed from *IAA7*‐edited plants and wild type. *IAA7‐*edited plants showed downward growth of flowers and pods (c) compared with wild type (b). (e) to (i), *IAA7*‐edited plants at seedling stage; j to o, *RGA*‐edited plants at seedling stage. (d) Wild type. Base‐edited T_0_ plants of *IAA7* (q to s) and *RGA* (t to w) showed dwarf phenotypes compared with wild type (p) at bloom stage. (x) Base‐edited plants of *RGA* exhibited semi‐dwarf and extreme dwarf compared with wild type at the pod maturation stage. Bar = 10 cm.

Dwarf mutant plants of *IAA7* maintained crinkled leaves with downward flowers and siliques (Figure 3a to d). All plants with base mutation in the conserved motif of *IAA7* displayed significantly decreased plant height at both seedling and maturation stages (Figure 3e to i and q to s). At the seedling stage, leaves of the dwarf mutant were severely crinkled and down‐curved (Figure 3e to i). Plants with four homoeologs edited showed extremely small architecture, growth stunt and were severely late flowering. These results were consistent with previous ones that showed mutation of conserved region of *IAA7* genes leading to dwarf phenotype. Plants with base mutation in the conserved motif of *RGA* showed reduced plant height at seedling stage (Figure 3j to o). At maturity, the homozygous mutants in C09, including *RGA‐5*, *18, 22* and 37, exhibited semi‐dwarf compared with that of wild type (Figure 3t to w). These plants had shorter main inflorescences and internodes with lower first primary branch positions. Mutated plants also showed semi‐dwarf phenotype at pod maturation stage (Figure 3x). Mutants with deletion in C09 of *RGA* displayed even shorter stature than that of other base‐editing plants (Figure 3x). Plants *RGA‐18* and *RGA‐22* exhibited semi‐dwarf phenotype with more branches (Figure 3x). Effective utilization of these semi‐dwarf mutants is critical for improving plant lodging resistance and yield performance.

### High‐throughput deep sequencing of base‐edited plants

**Figure 4:**
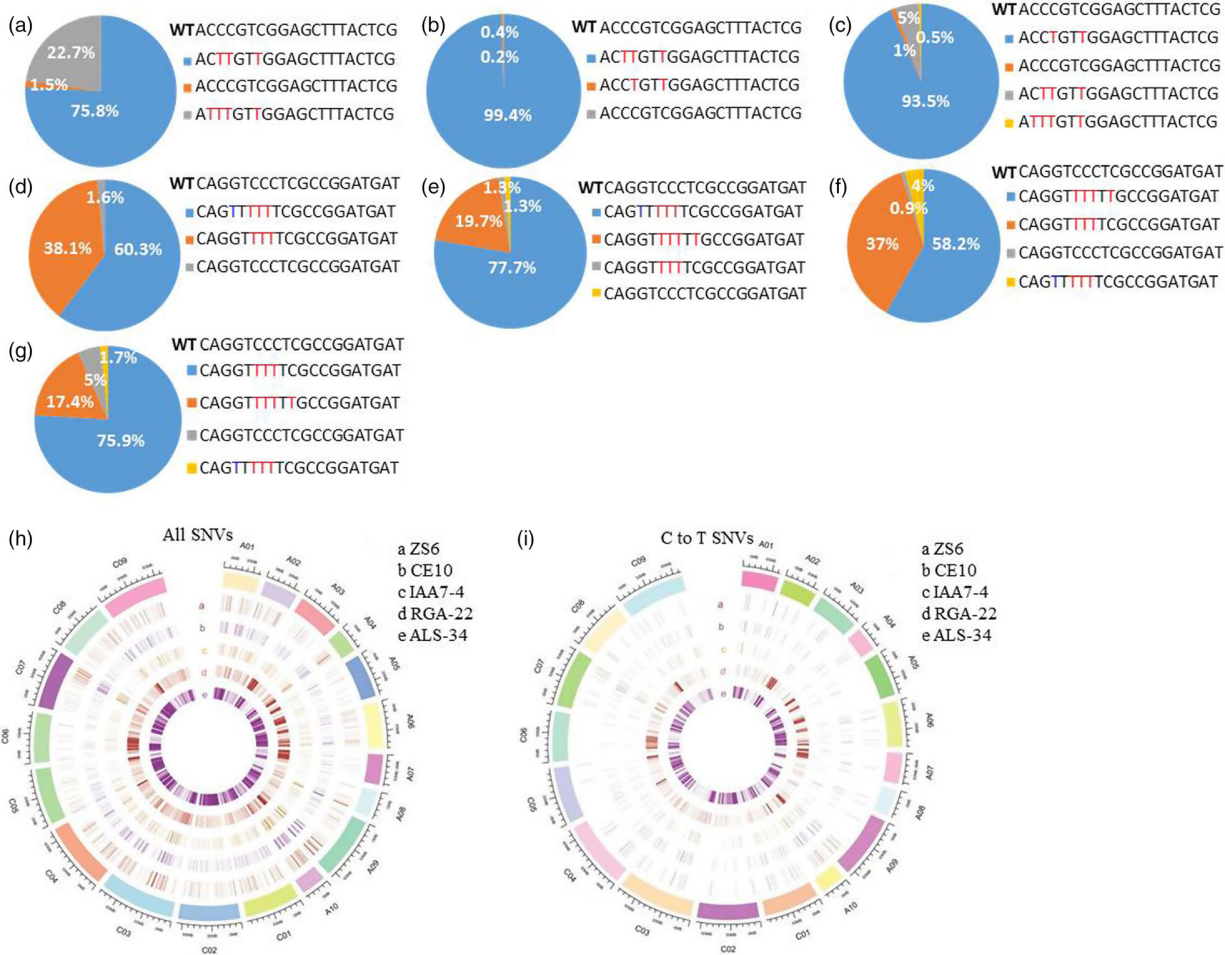
Target mutation analysis and SNP identification by deep sequencing. a to g, Base‐editing efficiency calculated by deep sequencing. Substitution efficiency was calculated by the ratio of base‐editing reads to the total reads. a to g, represented target sequence analysis for *RGA5*‐C9 (a), *RGA22*‐C9 (b), *RGA49‐*C9 (c), *ALS25*‐A1 (d), *ALS25*‐C1 (e), *ALS29*‐A1 (f) and *ALS29*‐C1 (g). Number represented ratio of base‐editing reads to total reads. C to T and G to T conversions were highlighted with red and blue colour, respectively. (h) and (i) represented all SNVs and C to T SNVs detected in plants transformed with A3A‐PBE elements without sgRNA, base‐edited plants and wild type on the 19 chromosomes in *B*.* napus* genome, respectively. Negative plant generated with same genetic transformation was used as control to perform SNP analysis.

As deep sequencing is very reliable for detection of different kinds of mutations with high‐throughput, we then performed barcode‐based sequencing of several independent transgenic plants from different target sites. PCR products amplified from the designed target region were purified to perform next‐generation sequencing. More than 200 000 reads were obtained from each PCR sample. For each sample, reads were filtered by barcodes and mapped to target genomic sequences, respectively. Two C to T substitution types within the editing window of *RGA5* at C09 chromosome were identified from 276,193 reads of *RGA5* plants, which accounted for 75.8% and 22.7%, respectively (Figure 4a). The ratio of sequence without any C to T substitution only accounted for 1.5% (Figure 4a). For another two edited plants *RGA22* and *RGA49*, the ratio of edited sequence was more than 93% (Figure 4b and c). We then analysed and compared editing efficiency of *ALS25* and *ALS29* in A and C chromosomes. The ratio of two edited sequences was 60.3% and 38.1% of *ALS25* in A chromosome (Figure 4d). About 78% and 20% editing efficiency was observed for two sequence types of *ALS25* in C chromosome (Figure 4e). The total ratio of edited sequences of *ALS29* was more than 92% in A and C chromosomes, respectively (Figure 4f and g). We also identified particular G to T conversion at C4 site in the target site of *ALS25‐A1* and *ALS25‐C1*, which is in accordance with the Sanger sequencing results (Figure 4d and e). As oilseed rape is an allotetraploid with A and C subgenomes, we speculated that different editing efficiency might exist among genes of the two subgenomes. However, the deep sequencing data indicated that there was no obvious bias of base editing between A and C genomes.

### Off‐target analysis

As our data revealed that A3APBE could generate considerable high‐editing efficiency in oilseed rape, it is crucial to know the off‐target effect of this system. Potential off‐target sites were predicted by CRISPR‐P software online (Xie *et al*., 2017). Potential off‐target sites that contain less than 5 mismatches with target sequence for three sgRNAs were arranged by the score values (Tables S5‐S7). Three most likely off‐target sites with high score value were selected, and the base‐editing frequency of these sites was analysed in edited plants for *ALS*, *RGA* and *IAA7* genes, respectively. PCR products covered for each sgRNA were amplified by specific primers and then subjected to Sanger sequencing directly. Sequencing data revealed that no difference of these potential off‐target sites between base‐edited and wild‐type plants (Table S11). These results revealed that the A3A‐PBE system may have low off‐target mutation efficiency in oilseed rape. However, off‐target mutation has been reported both in tomato and human genomes (Kim *et al*., 2019; Shimatani *et al*., 2017). Moreover, recent study showed that BE3 and HF1‐BE3 induced substantial genome‐wide off‐target mutations which were not located in the predicted off‐target sites after performed whole‐genome sequencing (WGS) analysis in rice (Jin *et al*., 2019). We then conducted whole‐genome sequencing for the edited plants to comprehensively investigate potential off‐target sites.

Whole‐genome sequencing was performed with at least 33 × sequencing depth for the base‐edited plants from three different sgRNAs, one negative plant (NE) generated from transformation, wild‐type ZS6 and one plant transformed with base editor without sgRNA (CE10). Overall alignment rate of Sequence data was more than 99%, and the coverage rate was more than 91% (Tables S8 and S9). Negative plant (NE) from genetic transformation without T‐DNA integration was used as control to conduct SNP and Indel analysis. The results showed that high level of SNPs was determined in two edited plants *ALS‐34* and *RGA‐22*, with a total of 614,226 and 216,439 SNP, respectively (Figure 4h and i, Table 2). However, low level of SNP variation was detected in *IAA7‐4,* CE10 plants and wild type, with 1110, 1287 and 925 SNPs, respectively (Table 2). SNPs were mostly identified to be located in the exon and intron regions (Table 3). We also discovered that the percentage of C to T transition was almost the same as A to G variation (Figure 4h, and i, Table 2). Meanwhile, about 113 762 and 90 449 Indels were identified for *ALS1‐34* and *RGA‐22*, respectively, whereas great lower level of Indels have been detected in other three plants (Table 2). We randomly selected 20 sites with SNP and 8 sites with Indel variation to perform Sanger sequencing of the three edited plants. About seventeen sites with SNP and 7 Indel variations were consistent with the whole‐genome sequencing result for each edited plants. Recent study demonstrated that cytosine base editors (BE3) induced substantial genome‐wide off‐target mutations with mostly the C to T variation (Jin *et al*., 2019). The average number of SNPs in BE3 or HF1‐BE3 without sgRNAs was also higher than those found in control plants (Jin *et al*., 2019). However, low level of SNP variation was detected both in *IAA7‐4* and CE10 plants, suggested that SNP variation caused by A3A‐PBE may correlated with sgRNA selection. Meanwhile, our study showed that the percentage of C to T transition was almost identical to that of A to G variation in the base‐edited plants (Table 2). Therefore, the A3A‐PBE may result in different levels of SNPs in oilseed rape genome and the degree is probably correlated with sgRNA selection. Occurrence of C to T conversion at whole‐genome level may be caused by increased expression level of deaminase and UGI (Jin *et al*., 2019). Recent study showed that ABEs could also cause widespread off‐target at the RNA level in mammalian cells (Kim *et al*., 2019). The off‐target effect could be resolved by developing more precise CBE with different cytosine deaminases and Cas nuclease fusions (Suhas *et al*., 2020).

**Table 2:** SNPs and Indel analysis for all samples

Samples	All_SNPs	All_Indels	C to T	A to G
Z6 VS CK	925	212	151	119
CE10 VS CK	1287	308	226	158
IAA7‐4 VS CK	1110	243	206	134
ALS34 VS CK	614 226	113 762	90 449	88 643
RGA22 VS CK	216 439	53 333	30 595	31 222

**Table 3:** Location of all SNPs and Indels

Samples	Exon	Intron	5_Upstream	3_Upstream
Z6 VS CK	110	230	5	10
CE10 VS CK	176	304	9	23
IAA7‐4 VS CK	177	302	11	8
ALS34 VS CK	97 273	110 462	7835	8389
RGA22 VS CK	55 002	57 624	4303	4587

### Genetic transmission of base‐edited mutation from T_0_ to T_1_ generation

**Figure 5:**
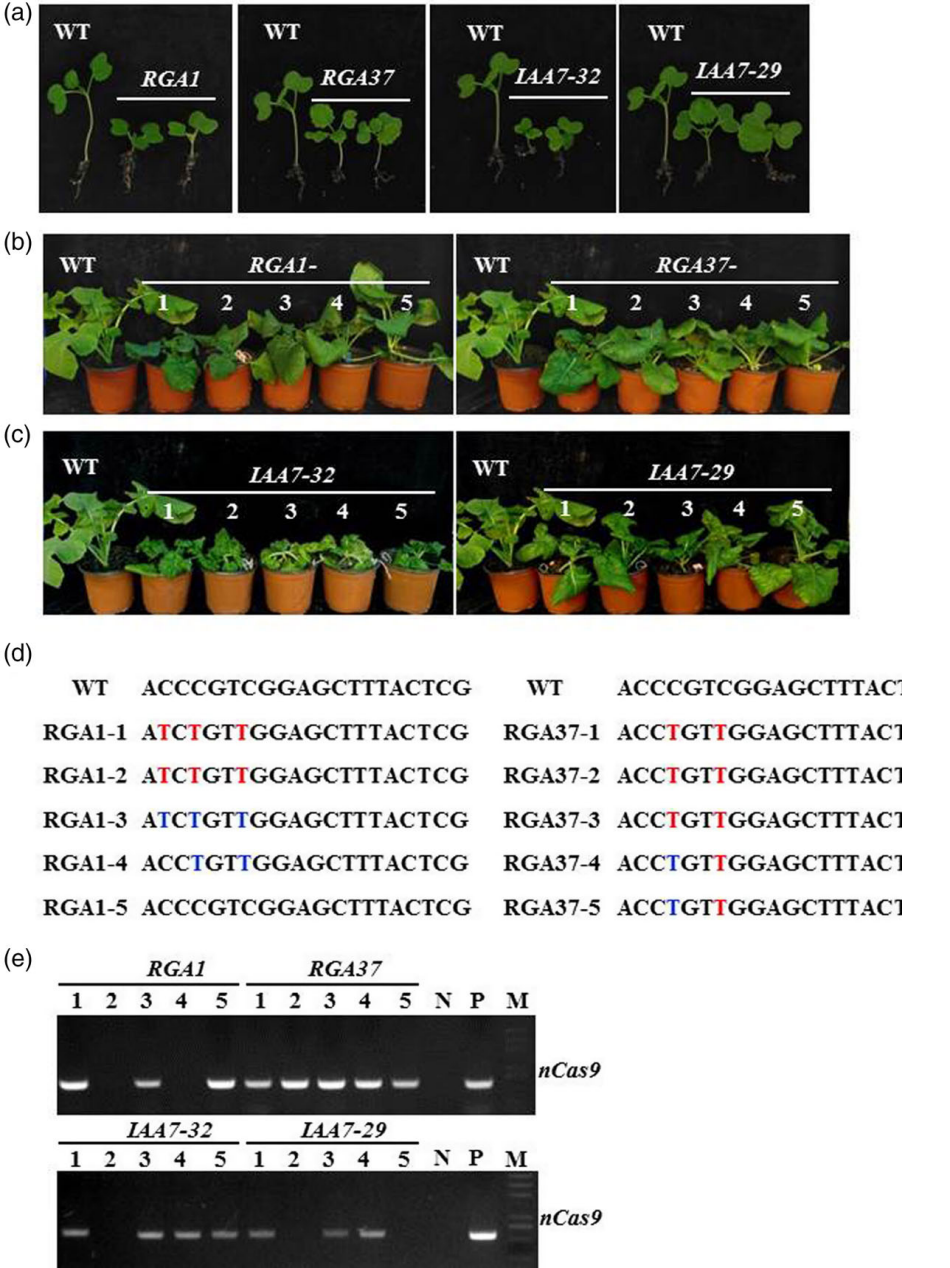
Phenotype of base‐edited plants of *RGA* and *IAA7* genes could be genetically inherited to T_1_ progenies. (a) Hypocotyl and cotyledons observation in *RGA* and *IAA7‐*edited plants. (b) T_1_ plants (1 to 5) from *RGA1* and *RGA37* T_0_ plants showed dwarf phenotypes compared with wild type. (c) T_1_ plants (1 to 5) from *IAA7‐32* and *IAA7‐29* T_0_ plants showed various dwarf phenotypes compared with wild type. (d) Genotype of independent T_1_ plants from *RGA*‐edited plants. Base‐editing sites with homozygous and heterozygous mutation are highlighted with red and blue colour, respectively. (e) PCR assay to examine foreign fragment with *nCas9* primers in T_1_ generation and WT. N, wild type; P, positive control; M, marker.

Different T_1_ families from independent T_0_ plants of *ALS1*‐edited plants were demonstrated to confer sulphonylureas herbicide resistance. To further evaluate the inheritance of base‐editing transgenic plants, T_1_ plants generated from T_0_ *RGA‐* and *IAA7‐*edited plants were also selected for further analysis. One week after germination, several seedlings of *RGA‐* and *IAA7‐*edited plants showed shorter hypocotyl with normal leaves and cotyledons compared with wild type (Figure 5a). At four‐ and six‐leave stages, a few of T_1_ seedling of *RGA‐1* still exhibited dwarf phenotype as T_0_ plants, whereas the negative plants (*RGA‐1‐5*) without base editing at target site displayed normal plant height (Figure 5b and d). Homozygous mutation plants (*RGA‐1‐1* and *RGA‐1‐2*) exhibited even shorter than heterozygous mutation plants (*RGA‐1‐3* and *RGA‐1‐4*) (Figure 5b and d). *RGA* homozygous mutation plants with two amino acids changed (*RGA‐37‐1,* −*2,* −*3*) showed shorter than plants with only one amino acid changed (*RGA‐37‐4* and −*5*; Figure 5b and d). We also observed that T_1_ plants generated from T_0_ plants of *IAA7‐32* and *IAA7*‐*29* displayed various dwarf phenotypes with crinkled leaves at seedling stage (Figure 5c). C to T substitution at the sgRNA target in different homoeologs was detected in T_1_ independent plants from both *IAA7‐32* and *IAA7‐29* by Sanger sequencing (Table S10). Thus, it was confirmed that base editing can be inherited from T_0_ plants (Figure 5d, Table S10). Previous studies showed that different homologs of *BnaIAA7* have divergent functions in plant height regulation (Cheng *et al*., 2019; Zheng *et al*., 2019). Mutation in the GWPPV motif of *BnaA3*.*IAA7* has great potential to yield heterosis by improving plant architecture with decreased plant height and branch angle (Li *et al*., 2019). Thus, isolation of plants with mutations at different homoeologs of *IAA7* or *RGA‐*edited plants will generate various dwarf mutant germplasm valuable for developing varieties for mechanic production in oilseed rape.

### Isolation of base‐edited plants without T‐DNA insertion element

Mutant lines with base editing at different target sites were self‐pollinated. To achieve plants with base editing without transgenic element in the rapeseed genome, we further performed PCR assay to detect T_1_ plants by *NPTII* and Cas9 primers. Plants without nCas9 gene elements were identified from *RGA1* and *IAA7‐32* T_1_ plants with base editing in the target sites (Figure 5e). These T‐DNA‐free plants exhibited dwarf or semi‐dwarf phenotypes. Meanwhile, we also isolated plants without T‐DNA elements from *ALS* mutants. Herbicide treatment showed that these T‐DNA‐free plants with desired base modifications conferred herbicide resistance (data not shown). All of these mutants could be potentially applied to confer sulphonylurea herbicides for better weed control or with better plant architecture.

## Conclusion

Collectively, we have generated mutants conferring herbicides tolerance and decreased plant height. These traits could be introduced into oilseed rape varieties and will greatly improve the mechanic production ratio. Our study revealed that A3A‐PBE base‐editing system could convert C to T substitution with an editing efficiency of more than 20% and wide editing window (C1 to C10) in oilseed rape. Indel mutations were verified in the target sites for all three sgRNAs for *ALS*, *RGA* and *IAA7* genes. Notably, large number of SNP variation was determined between the base‐edited and negative plants, and the degree of SNP variation may correlate with sgRNA selection. More other genes controlling important agronomic traits could be modified via this system and will be useful to facilitate the functional genomic research and varieties innovation in *B*.* napus*.

## Methods

### Vector construction

Human A3A cytidine deaminase fused with a Cas9 nickase (nCas9) and uracil glycosylase inhibitor elements were amplified from template plasmid p35S‐A3A‐PBE vector which was kindly provided by Prof. Caixia Gao, Institute of Genetics and Developmental Biology, Chinese Academy of Sciences. A3A‐PBE was digested by *Xho*I and *Kpn*I to generate the A3A‐PBE element and then ligated to pCambia2300. SgRNA transcription was driven by high‐transcription efficiency promoter of AtU6‐26 from Arabidopsis. The sgRNA expression cassette was amplified and cloned into the pCambia2300 using ClonExpressII One Step Cloning Kit (Vazyme, Nanjing, China). Primers used for vector construction are listed in Table S1.

### Agrobacterium‐mediated oilseed rape transformation

Genetic transformation mediated by Agrobacterium in oilseed rape was conducted as described before (Liu *et al*., 2014). Semi‐winter type oilseed rape variety Zhongshuang 6 (ZS6) was used as the transgenic receptor. Seeds were sterilized in 0.15% mercuric chloride solution for 15 min, washed with sterilized water for 3 times and then cultured in a chamber without light for 5 days. Elongated hypocotyls were cut into 5‐ to 8‐mm segments and immersed in Agrobacterium liquid medium. After transferred in co‐culture medium for 2 days, hypocotyl segments were then cultured in selection medium for about 3 weeks. Calli were transferred to regeneration medium and sub‐cultured for 3–4 times. Regenerated shoots were then moved to root medium, and plants with roots were transplanted into growth chamber.

### Mutation analysis for the target site by Sanger sequencing

Total genomic DNA was extracted from wild‐type ZS6 and transgenic plants using CTAB‐mediated method. Primers for *nCas9* and *NPTII* genes were applied for confirmation of transgenic plants. Specific primers were designed for different gene homoeologs after sequence alignment. PCR products were firstly sequenced by Sanger sequencing directly, and the positive amplicons were then ligated into pTOPO‐Blunt Cloning Kit vector (Aidlab biotechnologies, Beijing, China) and transformed into *E*. *coli* strain DH5α. After checked by PCR amplification, positive clones were selected for DNA Sanger sequencing.

### Targeted deep sequencing analysis

Barcode tags with a pair of 6‐base length were added to the end of primers to amplify the target sequences. For each independent sample, the corresponding primers with barcode were employed to perform PCR amplification. All the PCR products were mixed together with equal amounts and purified by purification kit (Tiangen, Beijing, China). DNA library was constructed according to the instruction (NEB DNA Library Prep Kit) and then applied for pair‐end sequencing of 150 bp by HiSeq X‐Ten of Illumina system (San Diego, California). Sequence quality and adaptor trimming were conducted by SOAPnuke 1.4 (BGI, Shenzhen, China). Raw data were analysed by Trimmomatic software (version 0.32, MINLEN:75) to remove low‐quality reads (Bolger *et al*., 2014). Data of target point mutation were sorted according to the specific barcode primers. Substitution frequency (C to T) was calculated for each sequence.

### Off‐target analysis

Potential off‐target sites were predicted by online tool CRISPR‐GE (Xie *et al*., 2017). Sequences with 1 to 5 bp mismatches to the target sites in the *Brassica napus* genome were selected as potential off‐target sites. The potential off‐targets were amplified by specific primers and used to perform Sanger sequencing directly. Data were investigated similar to target mutation site analysis.

### Whole‐genome sequencing analysis

Three base‐edited plants from *ALS*, *RGA*, *IAA7* genes, one wild‐type and one negative plant generated through the genetic transformation process but with no T‐DNA integration were selected to perform deep sequencing via 30 × depth using HiSeq X‐Ten of Illumina system (San Diego, California). SNP and Indel variations between base‐edited plants and negative plant control were identified by independent variant‐calling programmes. A few of sites were randomly selected to conduct Sanger sequencing to confirm SNP and Indel variation. Target base editing was also confirmed through the whole‐genome sequencing.

## Conflict of interest

The authors have declared that no competing interests exist in this manuscript.

## Authors’ Contributions

HTC, MYH and QH designed and carried out the research. MYH and HTC performed the genetic transformation and genotype analysis. WWX, BLD and MDS conducted deep sequencing. HTC, MYH and HW analysed the phenotype. HTC wrote the manuscript. QH, RJZ, CL and JL revised the manuscript. All authors read and approved the manuscript.

## Supporting information


**Table S1** Primers used in this study.

**Table S2** Base editing profile at *ALS* target site.
**Table S3** Base editing profile at *RGA* target site.
**Table S4** Base editing profile at *IAA7* target site.
**Table S5** Off target analysis for *ALS* gene in *Brassica napus*.
**Table S6** Off target analysis for *IAA7* gene in *Brassica napus*.
**Table S7** Off target analysis for *RGA* gene in *Brassica napus*.
**Table S8** Statistics of sequencing data and quality analysis.
**Table S9** Statistics of alignment of sequencing data.
**Table S10** Genotype of different lines from *IAA7‐32* and *IAA7‐29*.

**Table S11** Detection of most like off‐target site by Sanger sequencing.

**Table S11** Verification of potential off‐target of ALS by Sanger sequencing.

## Data Availability

Deep sequencing data of target site and potential off‐target site have been submitted to the NCBI Sequence Read Archive (SRA) with accession number (SRR11442836, SRR11440956, SRR11440947, SRR11440948, SRR11401918 and SRR11401888).
