# Supplementary material for: Base editing with high efficiency in allotetraploid oilseed rape by A3A‐PBE system
Source: Plant Biotechnol J. 2020 Aug 4;19(1):87–97. doi: 10.1111/pbi.13444 (PMC7769242; doi:10.1111/pbi.13444)
Supplement: Supplementary file 2 — Table S2 Base editing profile at ALS target site. Table S3 Base editing profile at RGA target site. Table S4 Base editing profile at IAA7 target site. Table S5 Off target analysis for ALS gene in Brassica napus. Table S6 Off target analysis for IAA7 gene in Brassica napus. Table S7 Off target analysis for RGA gene in Brassica napus. Table S8 Statistics of sequencing data and quality analysis. Table S9 Statistics of alignment of sequencing data. Table S10 Genotype of different lines from IAA7‐32 and IAA7‐29. [file PBI-19-87-s002.docx]

**Table S2** Base editing profile at *ALS* target site

| Plant number | A01.ALS | | C01.ALS | |
| --- | --- | --- | --- | --- |
|  | Base-editing position Indel | | Base-editing position Indel | |
| ALS3 | 6.7.8.10 C→T | N | N | N |
| ALS4 | N | N | 6.7.8.10 C→T | N |
| ALS7 | N | N | 6.7.8.10 C→T | N |
| ALS10 | 7.8.10 C→T | N | N | N |
| ALS13 | 6.7.8.10 C→T | N | 6.7.8.10 C→T | N |
| ALS18 | 6.7.8.10 C→T | N | 6.7.8.10 C→T | N |
| ALS22 | 1.6.7.8.10 C→T | N | 1.6.7.8.10 C→T | -19nt |
| ALS25 | 6.7.8 C→T | N | N | N |
| ALS29 | 6.7.8 C→T 4 G→T | N | 6.7.8.10 C→T | N |
| ALS34 | 6.7.8 C→T | N | 6.7.8.10 C→T | N |
| ALS36 | 6.7.8.10 C→T | N | 6.7.8.10 C→T | N |

**Table S3** Base editing profile at *RGA* target site

| Plant number | C09.RGA | | A09.RGA | | |
| --- | --- | --- | --- | --- | --- |
|  | Base-editing position Indel | | Base-editing position Indel | | |
| RGA1 | 4.7 C→T | -5nt | | N | N |
| RGA5 | 3.4.7 C→T | N | | N | N |
| RGA13 | 4.7 C→T | -14nt | | N | N |
| RGA18 | 3.4.7 C→T | N | | N | N |
| RGA22 | 3.4.7 C→T | N | | N | N |
| RGA25 | 4.7 C→T | N | | N | N |
| RGA27 | 3.4.7 C→T | -15nt | | N | N |
| RGA31 | 3.4.7 C→T | -15nt | | N | N |
| RGA37 | 4.7 C→T | N | | 5 G→T 7 C→T 14 T→C | N |
| RGA48 | 4.7 C→T | N | | N | N |
| RGA49 | 2.3.4.7 C→T | -11nt | | N | N |
| RGA50 | 2.3.4.7 C→T | N | | N | N |
| RGA55 | 2.3.4.7 C→T | N | | 5 G→T 7 C→T 14 T→C | N |
| RGA58 | 2.3.4.7 C→T | N | | N | N |
| RGA59 | 2.3.4.7 C→T | N | | N | N |
| RGA62 | 3.4.7 C→T | N | | N | N |

| Plant number | A03.IAA7 | | A05.IAA7 | | C01.IAA7 | | C05.IAA7 | |
| --- | --- | --- | --- | --- | --- | --- | --- | --- |
|  | Base-editing position Indel | | Base-editing position Indel | | Base-editing position Indel | | Base-editing position Indel | |
| IAA4 | 3.4.6.7 C→T | N | 3.4.6.7 C→T | N | 4.6.7 C→T | N | 4.6.7 C→T | N |
| IAA6 | 3.4.6.7 C→T | N | 3.4.6.7 C→T | -15nt | 3.4.6.7 C→T | N | N | N |
| IAA10 | 3.4.6.7 C→T | N | 3.4.6.7 C→T | N | 3.4.6.7 C→T | N | 3.4.6.7 C→T | N |
| IAA11 | 3.4.6.7 C→T | N | 3.4.6.7 C→T | N | 3.4.6.7 C→T | N | 3.4.6.7 C→T | N |
| IAA16 | 3.4.6.7 C→T | N | 6.7 C→T | N | 3.4.6.7 C→T | -26 nt | 4.6.7 C→T | N |
| IAA18 | 3.4.6 C→T | -14nt | 3.4.6.7 C→T | N | 3.4.6.7 C→T | +1nt | 3.4.6.7 C→T | N |
| IAA23 | 3.4.6.7 C→T | N | 3.4.6.7 C→T | N | 3.4.6.7 C→T | N | 3.5.6 C→T | N |
| IAA27 | 3.4.6.7 C→T | N | 3.4.6.7 C→T | N | 3.4.6.7 C→T | -251nt | 3.4.6.7 C→T | N |
| IAA29 | 3.4.6.7 C→T | N | 3.4.6.7 C→T | -15nt | 3.4.6.7 C→T | N | N | N |
| IAA32 | 6.7 C→T | -4nt | 3.4.6.7 C→T | -4nt | 3.4.6.7 C→T | N | N | -20nt |

**Table S4** Base editing profile at *IAA7* target site
