# Supplementary material for: Base editing with high efficiency in allotetraploid oilseed rape by A3A‐PBE system
Source: Plant Biotechnol J. 2020 Aug 4;19(1):87–97. doi: 10.1111/pbi.13444 (PMC7769242; doi:10.1111/pbi.13444)

**Verification of potential off-target of ALS by Sanger sequencing**

Predicted off target site: CCGGTCGGACGCCGGATGAT


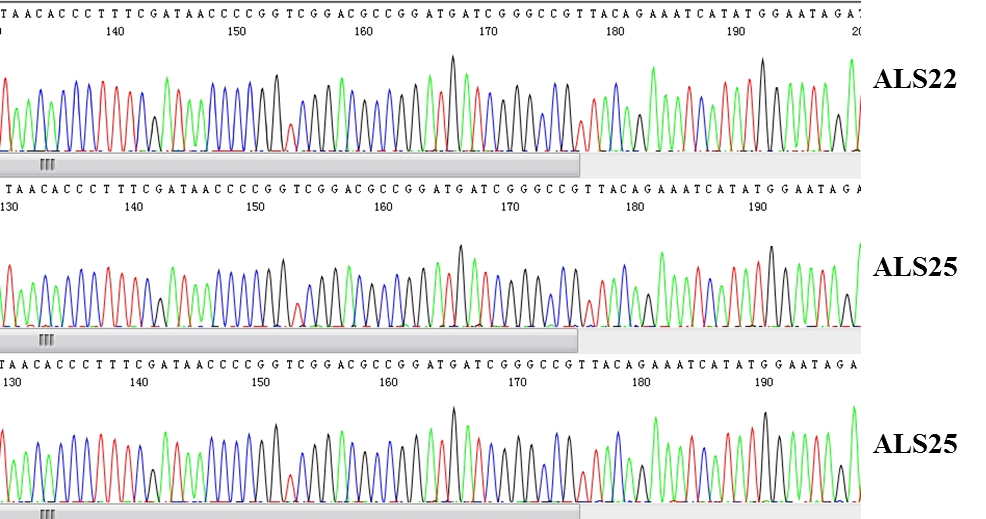


Predicted off target site: ATCATCCCGCGAGGGACCTG


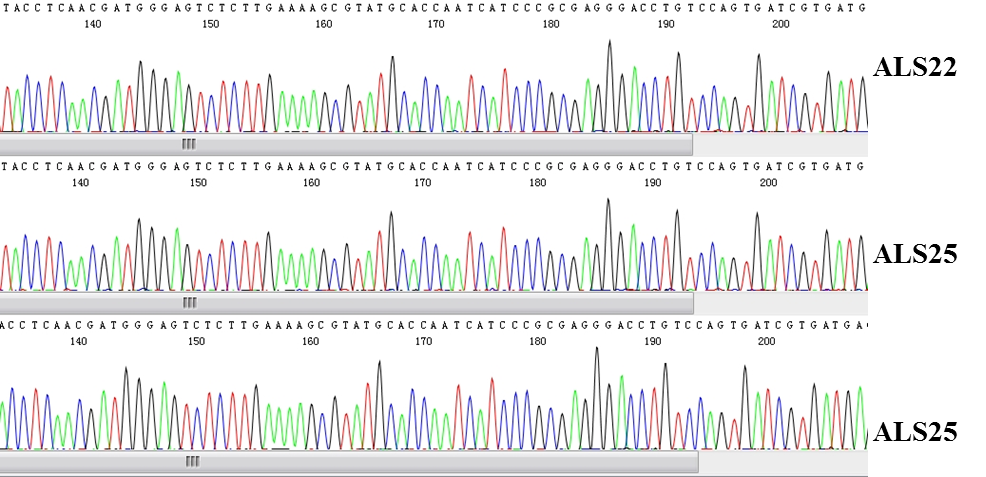


**Verification of potential off-target of RGA by Sanger sequencing**

Predicted off target site: ACCCCGCTGAGCTTTACTCG


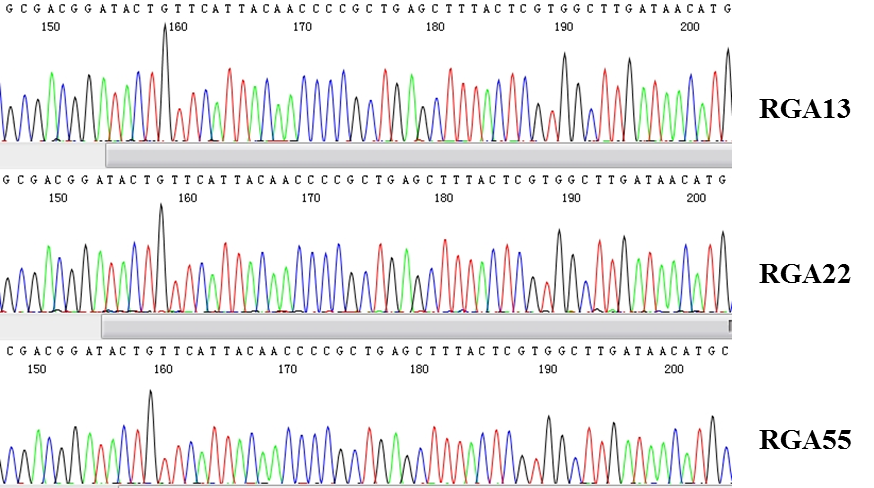


Predicted off target site: ATCCATTGGGGCTTTACCCG


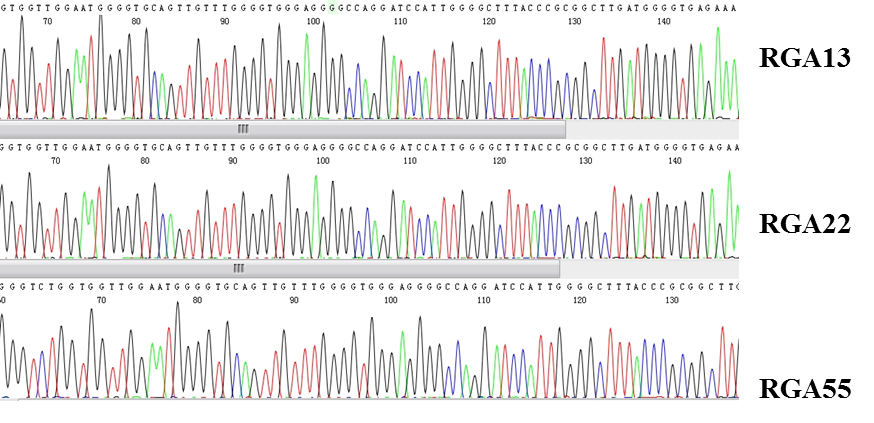


Predicted off target site: ACCCGTCGAAGCATAACTAG


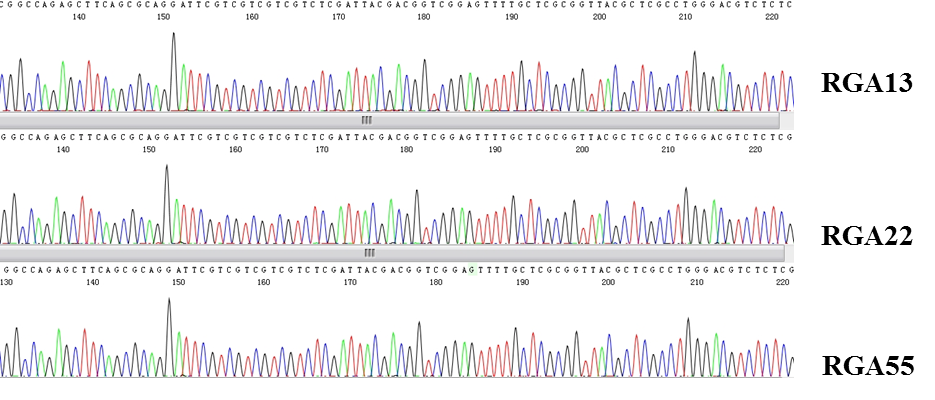


**Verification of potential off-target of IAA7 by Sanger sequencing**

Predicted off target site: GAAGTTCCTCACTGGAGGCA


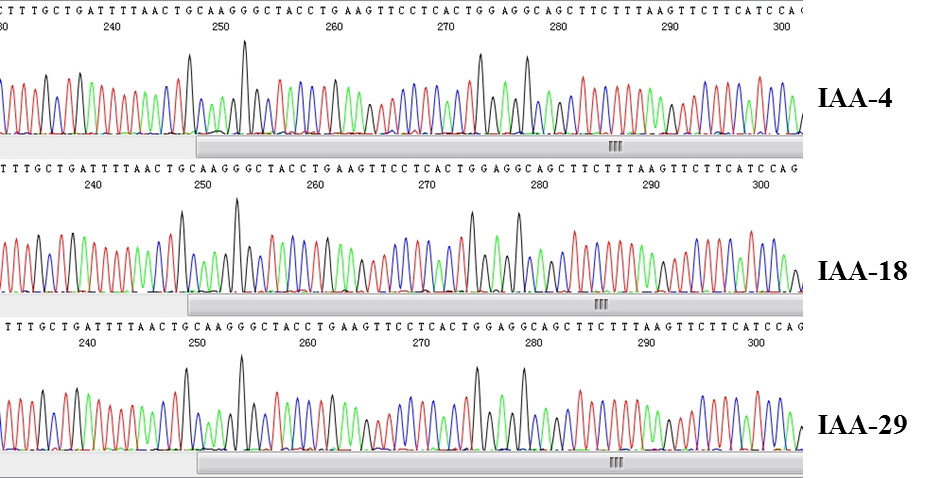


Predicted off target site: GGCGACCCGTGAGAAACAAA


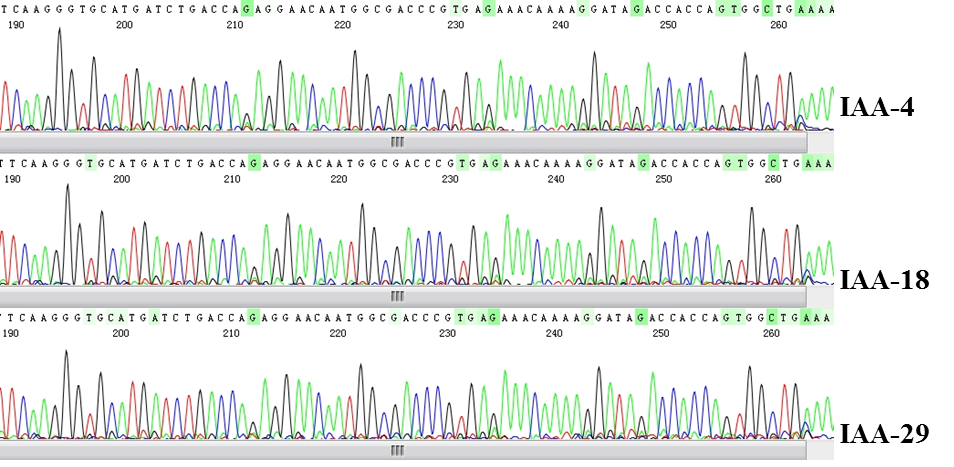


Predicted off target site: GTAGTTCCTCACCGGTGGCC


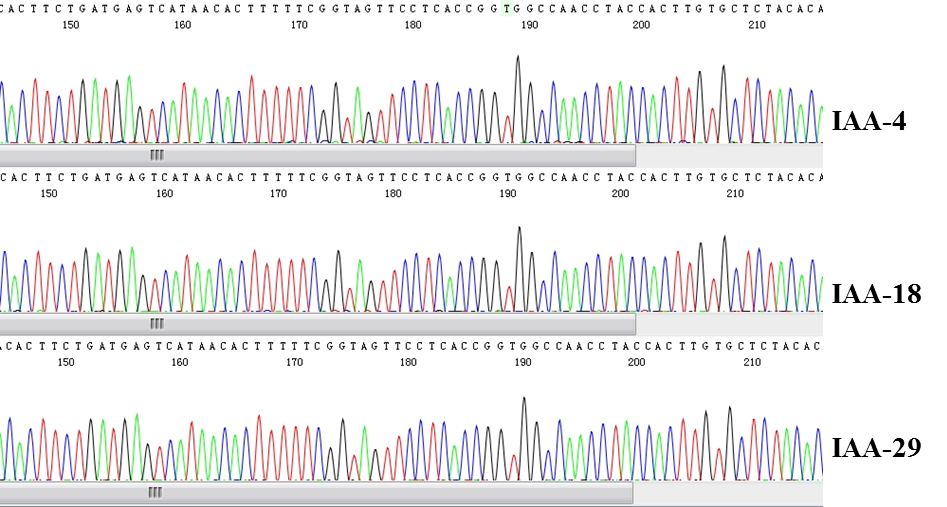

Supplement: Supplementary file 3 — Table S11 Detection of most like off‐target site by Sanger sequencing. [file PBI-19-87-s004.docx]
